# Immune response to pertussis vaccine in COPD patients

**DOI:** 10.1038/s41598-023-38355-8

**Published:** 2023-07-19

**Authors:** E. Feredj, A. Wiedemann, C. Krief, B. Maitre, G. Derumeaux, C. Chouaid, P. Le Corvoisier, C. Lacabaratz, S. Gallien, J. D. Lelièvre, L. Boyer

**Affiliations:** 1 Infectious Disease Department, AP-HP (Assistance Publique-Hôpitaux de Paris), Groupe Hospitalier Henri-Mondor/Albert Chenevier, 94010 Créteil, France; 2 INSERM U955, Equipe 16, IMRB (Institut Mondor de Recherche Biomédicale), Université Paris-Est-Créteil (UPEC), 94010 Créteil, France; 3 Vaccine Research Institute, 94010 Créteil, France; 4 Department of Physiology, APHP, Hôpital Henri Mondor, 94010 Créteil, France; 5 Department of Pulmonology, Centre Hospitalier Intercommunal, 94010 Créteil, France; 6 INSERM, Clinical Investigation Center 1430, Hôpital Henri Mondor, 94010 Créteil, France; 7 EA Dynamyc, Université Paris Est Créteil-École Vétérinaire de Maison Alfort, 94000 Créteil, France

**Keywords:** Microbiology, Health care, Vaccines, Immunology, Bacterial infection

## Abstract

Exacerbation triggered by respiratory infection is an important cause of morbidity and mortality in chronic obstructive pulmonary disease (COPD) patients. Strategies aiming to preventing infection may have significant public health impact. Our previous study demonstrated decreased immunological response to seasonal flu vaccination in COPD patients, questioning the efficiency of other vaccines in this group of patients. We performed a prospective, monocenter, longitudinal study that evaluated the humoral and cellular responses upon pertussis vaccination. We included 13 patients with stable COPD and 8 healthy volunteers. No difference in circulating B and T cell subsets at baseline was noted. Both groups presented similar levels of TFH, plasmablasts and pertussis specific antibodies induction after vaccination. Moreover, monitoring T cell immunity after ex-vivo peptide stimulation revealed equivalent induction of functional and specific CD4+ T cells (IFNγ, TNFα and IL-2-expressing T cells) in both groups. Our results highlight the immunological efficiency of pertussis vaccination in this particularly vulnerable population and challenge the concept that COPD patients are less responsive to all immunization strategies. Healthcare providers should stress the necessity of decennial Tdap booster vaccination in COPD patients.

## Introduction

Chronic obstructive pulmonary disease (COPD) is characterized by chronic lung inflammation that alters lung immunity and results into increased susceptibility to pulmonary infections^1^. Exacerbations are the main cause of morbidity and mortality in patients with COPD. They are responsible of accelerated decline in lung function, increased hospital admissions and mortality, and deteriorated quality of life^2^. Exacerbations are mostly triggered by respiratory infections^3^, therefore strategies aiming into preventing infection in COPD patients may have significant public health impact.

Impaired lung immunity is driven my multiple mechanisms including chronic damage to the airway epithelial barrier, defective mucus removal by ciliated epithelial cells^4^ and altered innate immune responses with diminished activation of alveolar macrophages, dendritic cells, neutrophils and natural killer cells^5,6^. Moreover, despite important influx of CD8^+^ T cells and the formation of tertiary lymphoid follicles in the lungs of COPD patients^7^, increased circulating numbers of exhausted PD1^+^ T cells^8^ and an immunosuppressive cytokine profile with the elevation of IL-10 and TGF-β^1^ potentially negatively impacting T cell function. Finally, several studies suggested that vaccines may be less efficient in COPD patients^9,10^.

*Bordetella pertussis* is a gram-negative pathogenic bacillus and the causative agent of pertussis, an acute respiratory infection, that can be prevented with vaccination using the acellular pertussis vaccine (aP)^11,12^. The past few years have witnessed a resurgence in pertussis, including in COPD patients, with dramatic consequences on lung function and mortality risks^13,14^. Among adults over 65 years old hospitalized for severe pertussis infection from 2011 to 2015 in the United States, 26.8% had history of COPD. However, the immune response and the efficacy of aP vaccination in COPD patients are poorly characterized. We therefore aim to analyze and evaluate the immune response following pertussis vaccination in patients with COPD.

## Patients and methods

We have investigated the immune response to acellular pertussis vaccination in COPD patients and healthy volunteers. We performed a prospective, monocenter, longitudinal study that evaluated the humoral and cellular responses upon vaccination. Patients with stable COPD and healthy volunteers were recruited from September 2018 to May 2019 at the pulmonary out-patient clinic of the Henri Mondor Hospital or Intercommunal Hospital of Créteil (France). Stable COPD patients as are defined as COPD patients with no history of exacerbation and/or upper or lower respiratory infection in the month preceding inclusion in the study. All included patients were eligible for combined diphtheria-tetanus-polio-pertussis vaccine (Tdap). Exclusion criteria included acute heart failure, malignancy in the past two years, ongoing pregnancy, immunosuppressive treatment or disease, and/or the presence of contraindication for vaccination following European guidelines. Healthy volunteers presented no symptoms of pulmonary disease and a normal spirometry. Participants in this study were administered a single dose of Tdap via intramuscular injection on day 0. The study involved four clinical visits scheduled at day 0, 7, 15, and 30 post-vaccinations. At each visit, blood samples were collected for analysis. The humoral response, specifically the analysis of Tfh cells and plasmablasts, was examined on day 0 (prior to vaccination) and day 7. Additionally, serum antibody titers against pertussis were measured using blood samples obtained on day 0 and day 30. To evaluate cellular immunity, T cell specific responses were assessed using intracellular cytokine staining. This analysis was performed 15 days after vaccination, following *ex-vivo* stimulation of peripheral blood mononuclear cells (PBMCs) with a *Bordetella pertussis* peptide pool. Supernatants from stimulated cells were also collected for further analysis. Statistical analysis were performed using Prism (GraphPad) version 8.4.0.

The study conforms to the principles outlined in the Declaration of Helsinki, received approval by the appropriate Institutional Review Board and was performed in accordance with the relevant regulations. All participants provided written informed consent before inclusion. The trial is registered as NCT 03804138 and has been approved by the 2018/53 Protection of Persons Committee Ile de France IV Institutional Review Board Agreement of US Department of Health and Human Services No. IRB 00003835.

## Results

We included 13 patients with clinically stable COPD and 8 healthy volunteers (Table 1). Age was similar in both groups (median, 64 years; interquartile range (IQR) 56.25 to 67.75 in healthy volunteers and 68 years; IQR 61 to 70 in patients with COPD). Males represented 6/8 individuals in healthy volunteers and 12/13 patients in the COPD group. The median number of pack-year smoking was 24; IQR 10 to 40 in healthy volunteers’ group and 55; IQR 25.75 to 69 in the COPD patients. Forced expiratory volume in 1 s (FEV1) was lower in patients with COPD (median 57%; IQR 42.5–66.5 versus 102%; IQR 100–104.5), as well as forced vital capacity (FVC) (median 77%; IQR 64.5–90.5 versus 101%; IQR 96–111), FEV1/FVC ratio (median 52%; IQR 49.5–64 versus 81%; IQR 76.5–88) and diffusing capacity of the lungs for carbon monoxide (DLCO) (median 60%; IQR 30–75 versus 91%; IQR 81.5–107.5).

**Table 1:** Main features in participants with COPD and in controls.

	Controls (n = 8)	COPD (n = 13)
Males/females	6M/2F	11M/2F
Age (years)	64 (56.25–67.75)	68 (61–70)
Pack year	24 (10–40)	55 (25.75–69)
FEV (% predicted)	102 (100–104)	57 (42.5–66.5)
FVC (% predicted)	101 (96–111)	77 (64.5–90.5)
FEV/FVC (% predicted)	81 (76.5–88)	52 (49.5–64)
DLCO (% predicted)	91 (81.5–107.5)	60 (81.5–107.5)

Data are median ± interquartile range (IQR).

*DLCO* diffusing capacity of the lung for carbon monoxide, *FVC* forced vital capacity, *FEV* forced expiratory volume.

**Figure 1:**
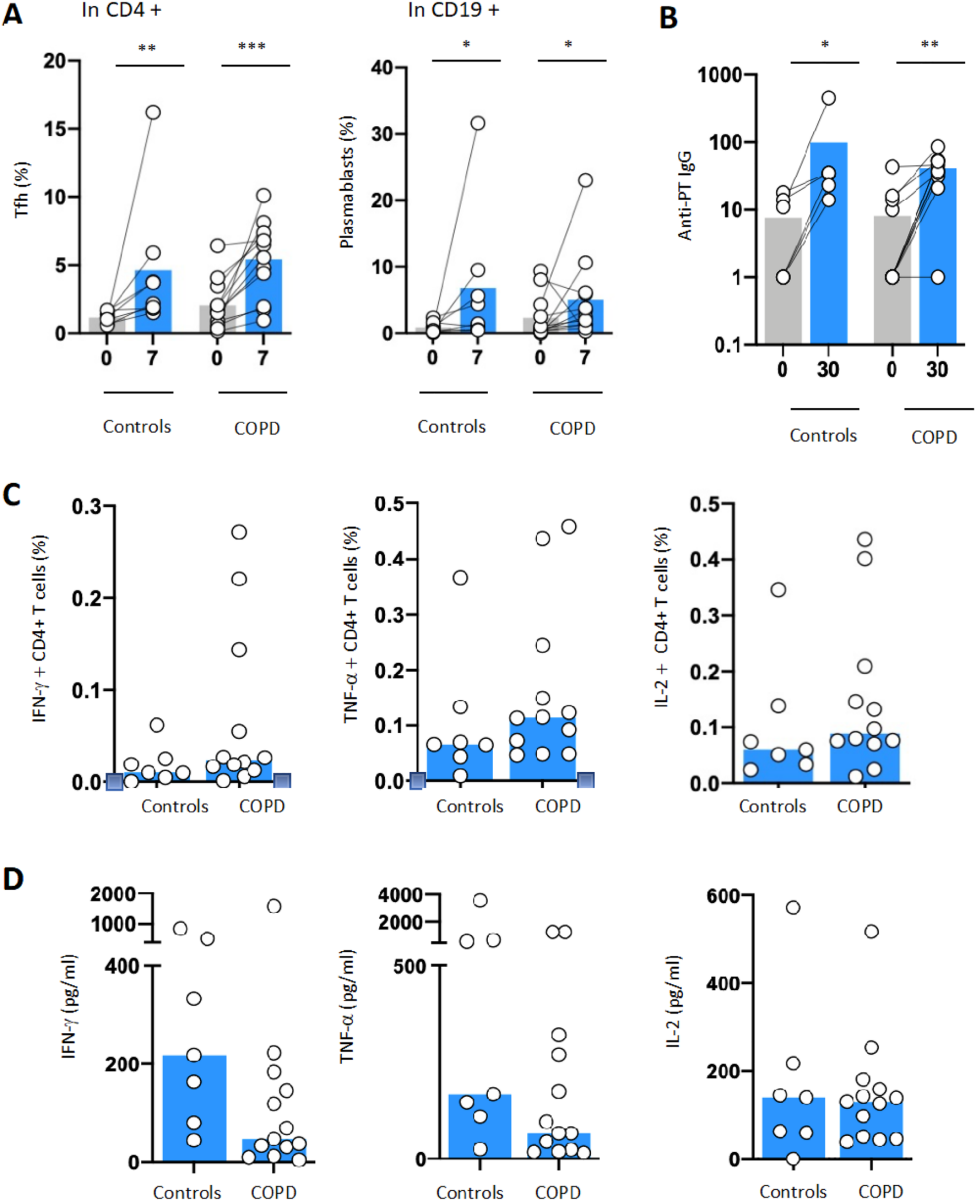
Humoral and cellular responses to *Bordetella pertussis* stimulation in patients with COPD compared to controls. To measure immune responses to Pertussis vaccine, blood from n = 8 control donors and n = 13 patients was withdrawn at baseline and at day 7 or 28 days upon vaccination. (**A**) TFH cells (CD3+ CD4+ CXCR5+ PD1+) and plasmablasts cells (CD19+ CD38+) were measured by flow cytometry (LSRII, BD Biosciences), at baseline (D0) and 7 days (D7) after pertussis vaccination in COPD patients (n = 12) and controls (n = 8). (**B**) Anti-pertussis circulating immunoglobulin G (anti-PT IgG) antibodies were measured using enzyme immunoassay at D0 and D7 in COPD patients (n = 11) and controls (n = 6) (Biomnis). (**C**) T cell specific responses were monitored with intracellular cytokine staining for IFN-γ, IL-2, TNF-α following ex vivo PBMC stimulation with Bordetella pertussis peptide pool (filamentous hemagglutinin [FHA], fimbriae 2/3 [Fim2/3], pertactin [PRN], and inactivated pertussis toxin [PT]) antigens) in patients with COPD (n = 12) and controls (n = 7). (**D**) Concentration of IFN-γ, IL-2, TNF-α (pg/mL) was measured in culture supernatants from COPD patients (n = 13) and controls (n = 13) using Luminex technology following manufacturer instructions (HCYTMAG-60K-PX41). Supernatants were collected on day 5 after stimulation with Bordetella pertussis peptide. Median values are shown and statistical significance was evaluated by Wilcoxon test; *P < 0.05; **P < 0.01; ***P < 0.001.

At baseline, we noted no significant difference in circulating B cell subsets, CD4+ and CD8+ T cell subsets and T-follicular Helper (TFH) cells (defined as CD4+ CXCR5+ ICOS+ PD1high) (Fig. 1A). Interestingly, there was a trend towards an increase in naïve B cells (CD19+ CD21+ CD27−), and a decrease in memory B cells (CD19+ CD27+ CD21+) in COPD patients as previously described (10). Moreover, CD4+ T cells expressed higher surface level of Programmed-death (PD)-1 at baseline in COPD patients. At day 7 after vaccination, we observed a significant increase in TFH and plasmablasts (CD19+ CD27+ CD38high) as compared to day 0 for Tfh and plasmablast (respectively P < 0.001 and P < 0.05) reflecting the induction of humoral response in both groups (Fig. 1A). Accordingly, pertussis specific antibody levels were similarly increased in both groups 30 days after vaccination (Fig. 1B). Regarding cellular immunity, 15 days after vaccination, frequency of pertussis-specific CD4+ T cells, quantified by staining for IFN-γ, IL-2 and TNF-α, was equivalent in both groups supporting the induction of T cell immunity (Fig. 1C). Analysis of supernatants from stimulated cells and confirmed that IFN-γ, IL-2 and TNF-α secretion was equivalent in both group (Fig. 1D).

## Discussion

Our data support vaccine efficiency to pertussis vaccination in COPD patients, in contrast to our previous influenza vaccination study that demonstrated an impaired humoral and cellular response to influenza vaccination^10^. While several hypothesis may explain this discrepancy, such as differences in vaccine design and adjuvant composition^15^, it is possible that in the specific case of influenza, re-exposure after an original exposure preferentially boosts the immune response against the original strain^16^, dictated by how the original exposure shaped the B and T cell repertoire^17^. This hypothesis, designated as the original antigenic sin (OAS) has been indeed reported for seasonal influenza vaccine^18^ with the observation that influenza strains encountered later in life boosted the humoral response to the original strain with the same subtype^19^. However, in the case of the Tdap vaccination for pertussis, several factors may contribute to a different immune response pattern. Firstly, the decreased incidence of pertussis infections due to successful vaccination programs has resulted in lower overall exposure to pertussis antigens. Furthermore, the Tdap vaccination schedule typically involves fewer doses, potentially reducing opportunities for immune priming and subsequent original antigenic sin effects.

Our study has limitation. It is a monocentric, prospective and exploratory study, with a limited number of included patients. Conclusions regarding some of the immunological and the clinical impact of pertussis vaccination in COPD patients are to be taken with caution and require further validation with larger cohorts.

In conclusion, our results highlight the immunological efficiency of pertussis vaccination in this particularly vulnerable population and challenge the concept that COPD patients are less responsive to all immunization strategies. Healthcare providers should stress the necessity of vaccination for pertussis with decennial Tdap booster vaccination in COPD patients.

## Data Availability

Data is available upon reasonable request to the corresponding author.

## References

[1] Bhat TA, Panzica L, Kalathil SG, Thanavala Y (2015). Immune dysfunction in patients with chronic obstructive pulmonary disease. Ann. Am. Thorac. Soc..

[2] Donaldson GC (2002). Relationship between exacerbation frequency and lung function decline in chronic obstructive pulmonary disease. Thorax.

[3] Papi A (2006). Infections and airway inflammation in chronic obstructive pulmonary disease severe exacerbations. Am. J. Respir. Crit. Care Med..

[4] Thorley AJ, Tetley TD (2007). Pulmonary epithelium, cigarette smoke, and chronic obstructive pulmonary disease. Int. J. Chron. Obstruct. Pulmon. Dis..

[5] Shaykhiev R, Crystal RG (2013). Innate immunity and chronic obstructive pulmonary disease: A mini-review. Gerontology.

[6] Berenson CS (2006). Impaired alveolar macrophage response to Haemophilus antigens in chronic obstructive lung disease. Am. J. Respir. Crit. Care Med..

[7] Brusselle GG, Joos GF, Bracke KR (2011). New insights into the immunology of chronic obstructive pulmonary disease. Lancet.

[8] Wilkinson TMA (2017). Immune checkpoints in chronic obstructive pulmonary disease. Eur. Respir. Rev..

[9] Poole PJ, Chacko E, Wood-Baker RWB, Cates CJ (2006). Influenza vaccine for patients with chronic obstructive pulmonary disease. Cochrane Database Syst. Rev..

[10] Parpaleix A (2017). Impaired humoral and cellular immune responses to influenza vaccination in chronic obstructive pulmonary disease patients. J. Allergy Clin. Immunol..

[11] https://vaccination-info.eu/en/disease-factsheets/pertussis.

[12] https://vaccination-info-service.fr/Les-maladies-et-leurs-vaccins/Coqueluche.

[13] Mbayei SA (2019). Severe pertussis infections in the United States, 2011–2015. Clin. Infect. Dis..

[14] Blasi F (2019). The unmet need for pertussis prevention in patients with chronic obstructive pulmonary disease in the Italian context. Hum. Vaccine Immunother..

[15] Nabel GJ (2013). Designing tomorrow’s vaccines. N. Engl. J. Med..

[16] Zhang A, Stacey HD, Mullarkey CE, Miller MS (2019). Original antigenic sin: How first exposure shapes lifelong anti-influenza virus immune responses. J. I..

[17] Fonville JM (2014). Antibody landscapes after influenza virus infection or vaccination. Science.

[18] Krammer F (2019). The human antibody response to influenza A virus infection and vaccination. Nat. Rev. Immunol..

[19] Miller MS (2013). Neutralizing antibodies against previously encountered influenza virus strains increase over time: A longitudinal analysis. Sci. Transl. Med..

